# The transferability of handwriting skills: from the Cyrillic to the Latin alphabet

**DOI:** 10.1038/s41539-021-00084-w

**Published:** 2021-02-23

**Authors:** Thibault Asselborn, Wafa Johal, Bolat Tleubayev, Zhanel Zhexenova, Pierre Dillenbourg, Catherine McBride, Anara Sandygulova

**Affiliations:** 1 CHILI Lab, EPFL, Route Cantonale, 1015 Lausanne, Switzerland; 2 Faculty of Engineering, UNSW, Sydney, NSW Australia; 3 Department of Robotics and Mechatronics, School of Engineering and Digital Sciences, Nazarbayev University, Nur-Sultan, Kazakhstan; 4 Department of Psychology, The Chinese University of Hong Kong, Hong Kong, China

**Keywords:** Education, Learning and memory, Motor control

## Abstract

Do handwriting skills transfer when a child writes in two different scripts, such as the Latin and Cyrillic alphabets? Are our measures of handwriting skills intrinsically bound to one alphabet or will a child who faces handwriting difficulties in one script experience similar difficulties in the other script? To answer these questions, 190 children from grades 1–4 were asked to copy a short text using both the Cyrillic and Latin alphabets on a digital tablet. A recent change of policy in Kazakhstan gave us an opportunity to measure transfer, as the Latin-based Kazakh alphabet has not yet been introduced. Therefore, pupils in grade 1 had a 6-months experience in Cyrillic, and pupils in grades 2, 3, and 4 had 1.5, 2.5, and 3.5 years of experience in Cyrillic, respectively. This unique situation created a quasi-experimental situation that allowed us to measure the influence of the number of years spent practicing Cyrillic on the quality of handwriting in the Latin alphabet. The results showed that some of the differences between the two scripts were constant across all grades. These differences thus reflect the intrinsic differences in the handwriting dynamics between the two alphabets. For instance, several features related to the pen pressure on the tablet are quite different. Other features, however, revealed decreasing differences between the two scripts across grades. While we found that the quality of Cyrillic writing increased from grades 1–4, due to increased practice, we also found that the quality of the Latin writing increased as well, despite the fact that all of the pupils had the same absence of experience in writing in Latin. We can therefore interpret this improvement in Latin script as an indicator of the transfer of fine motor control skills from Cyrillic to Latin. This result is especially surprising given that one could instead hypothesize a negative transfer, i.e., that the finger controls automated for one alphabet would interfere with those required by the other alphabet. One interesting side-effect of these findings is that the algorithms that we developed for the diagnosis of handwriting difficulties among French-speaking children could be relevant for other alphabets, paving the way for the creation of a cross-lingual model for the detection of handwriting difficulties.

## Introduction

Despite the increasing use of tablets and laptops in schools, handwriting has maintained its central position in school education systems. Handwriting remains a paramount skill to be mastered in school since it is the basis of many core educational activities such as taking notes, composing stories and self-expression^1–3^. Handwriting is a complex perceptual-motor task, as it involves attention, perceptual, linguistic and fine motor skills^4–9^. Hence, even in typically developing children, learning handwriting usually begins around the age of five (pre-school) and last around 10 years^10,11^. During this time, handwriting evolves initially on a qualitative level (legibility) and then on a quantitative level (speed)^12^.

Despite proper training, a significant number of children never reach a sufficient level of automation in handwriting. These handwriting difficulties affect around 10% of children^13,14^ in European countries. With the increasing cognitive demand from school work throughout their curriculum, these children become rapidly unable to face simultaneous efforts involving such items as handwriting, grammar, orthography, and composition, leading to an increase in fatigue, a decrease in self-esteem, and a general decrease in cognitive performance with the potential to impact both children’s behavioral and academical development seriously^8^. It is, thus, of prime importance to detect handwriting difficulties as soon as possible in order to apply the earliest remediation^15,16^.

Many tests allowing for the diagnosis of handwriting difficulties have been proposed in different languages and alphabets^13,17,18^. Most of the methods assess handwriting based on several predefined, specific criteria graded by an expert. Such is the case, for example, with the Concise Evaluation Scale for Children’s Handwriting (BHK), the gold standard test for diagnosing handwriting difficulties in several European countries^19^.

Many limitations come with the use of these tests. In addition to the subjectivity resulting from human scoring and time-consuming corrections, all of these tests are conducted using a pen/pencil and paper, meaning that their scoring is restricted to the analysis of the final, static handwriting product and does not consider or include any information about movement dynamics, which are crucial for the analysis of handwriting disorders^20–22^.

The development of digital tablets in the last decade has allowed us to tackle these problems since the dynamics of handwriting can now be assessed. Few handwriting tests for digital tablets have thus far been invented in different alphabets such as Latin or Hebrew^21,23,24^. In the model of interest for this study, Asselborn et al.^21^ extracted 53 features describing handwriting via different aspects and sorted them into four main categories, namely, static (which can possibly be measured with a pen/paper test), kinematic, pressure and tilt. These features were designed to capture low levels, almost physiological, aspects of handwriting (e.g. the micro-frequencies of shakiness) that are therefore independant of the shape of the letters and that could thus be transferred to other language and alphabets. In addition to reaching a remarkable accuracy in the binary diagnosis of severe handwriting difficulties (dysgraphia), the method can also characterize handwriting quality for the different features thanks to a comparison with normative children.

Digraphia (or bigraphism)^25^ refers to the use of two (or more) scripts for the same language^26^. It can be characterized into several types: synchronic (e.g. Serbo-Croatian or Hindi-Urdu^26^), diachronic/sequantial (e.g. change from Arabic to Latin script in Turkey or from Arabic to Latin to Cyrillic to Latin in Turkmenistan, Azerbaijan, or Uzbekistan and now to be performed in Kazakhstan by 2025^27^), concurrent (e.g. use of Kanji plus Hangul in South Korea), projected/functional (e.g. use of Pinyin as alphabetical representations for Chinese characters^26,28^), and computer-mediated (e.g. "Greeklish”^29^ or “Arabizi”^30^). Out of all these cases of digraphia, sequential or concurrent digraphia is the only case where it is required to develop handwriting proficiency in both scripts for the same population, especially in the stage of a transition. This is the case of Kazakhstan which, by 2025, will have abandoned the Cyrillic alphabet in favor of the Latin alphabet for writing in the Kazakh language. This political decision generates interest for this study.

Relatively little is known about the transfer of writing skills across scripts and it is extremely difficult to isolate the role of motor skills in this transfer, as compared to other cognitive and linguistic skills. Since handwriting combines visual, language, and motor dimensions, neuroimaging does not provide a clear account of cerebral substrates of handwriting. In their meta-analysis based on 18 studies of patients who lost handwriting skills, Planton et al.^31^ concluded that no less than 12 areas were involved, even if mainly located in the left frontal and superior parietal regions. This complexity also concerns empirical studies. For example, one study of 10-year-old Hong Kong Chinese children found a correlation of 0.64 between dictation performance in Chinese and spelling ability in English^32^, which might reveal some transfer across scripts. However, spelling involves a variety of linguistic skills apart from handwriting itself, being sensitive to children’s phonological sensitivity, vocabulary knowledge, or even general reasoning, among others. A set of studies have explored writing across different scripts as a relatively pure measure of handwriting alone. The studies focused on Chinese children from kindergarten and primary school in Mainland China and Hong Kong. The original intention was to document how copying of 2-dimensional unfamiliar print is associated with children’s word reading and writing in Chinese. Chinese children’s copying of Korean Hangul, Hebrew and Vietnamese (which is written in the Roman script but requires a variety of complex and unfamiliar diacritics) were tested across different studies. Such copying in novices represents a kind of visual-motor ability, separate from familiar cognitive-linguistic skills. The measures of unfamiliar print copying were scored differently based on the requirements of each script. Across studies^33–37^, children’s performances with these unfamiliar scripts, either individually or as a copying factor, were significantly associated with and sometimes uniquely (apart from other cognitive-linguistic skills) predictive of their native Chinese dictation skills. Moreover, measures of copying performance of one unfamiliar script with another were also moderately associated^33,35^. Finally, in one study of kindergartners^37^, copying of unfamiliar script was correlated 0.42 with the well-established measure of visual-motor skill called the Beery VMI^38^. Collectively, these studies suggest that handwriting skills across scripts may share a core coherency that taps various aspects of handwriting that might transfer, including, but not limited to, attention to fine detail, spacing, pressure, and density.

Kazakhstan recently adopted a state program for the development and functioning of languages for 2011–2020. This new tri-lingual education policy aims for the Kazakhs to develop fluency in three languages: Kazakh, Russian and English. Additionally, a motion to transfer the Kazakh language from Cyrillic to the Latin alphabet was approved by the Kazakh authorities in October 2017^27^. While there are clear reasons for these reforms, there are numerous risks surrounding the transfer, including risks of increasing inequalities in educational services (e.g., preferences for Russian-speaking schools), causing illiteracy in adults in their native language and bringing about disinterest and lack of motivation in reading and writing in Kazakh among Kazakh and non-Kazakh children and adults.

**Fig. 1:**
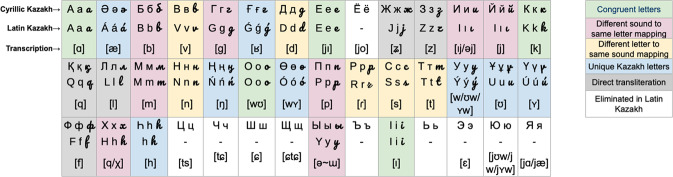
Similarities and differences between Cyrillic and Latin Kazakh alphabets. Each letter is presented in three forms: print capital, print small, and cursive small forms. Also included the phonetic form of each letter.

In fact the switch across alphabets is not min: while there are 42 letters in the old Cyrillic Kazakh, the new Latin Kazakh includes only 32 letters, out of which only 23 letters are also found in English^39^. Despite the fact that both alphabets (Cyrillic and Latin) were derived from the Greek alphabet, there is only a handful of letters that are phonologically congruent across alphabets (such as “A”, “O”, “E”, “I”, and ”K”), decoded into the same sound either in Cyrillic or Latin. Indeed, several letters show different sound to same letter mapping across alphabets (such as these Cyrillic to Latin mappings: “P” [r] - “P” [p], “B” [v] - “B” [b], “H” [n] - “H” [h], etc.), and different letter to same sound mapping across alphabets (such as these Cyrillic to Latin mappings: “” [b] - “B” [b], “Γ” [g] - “G” [g], “C” [s] - “S” [s], “ “K” [q] - Q [q], etc.) There are also eight unique Kazakh sounds that are represented with acutes that are not found in the English alphabet, whereas ten Cyrillic letters no longer appear in the Latin version. Figure 1 demonstrates similarities and differences between the alphabets. As shown, only five letters are directly transliterated (such as these Cyrillic to Latin mappings: “Φ” [f] - “F” [f], “” [l] - “L” [l], “[d]” - “D” [d], etc.) Therefore, one’s knowledge of Latin English might help with a few Latin Kazakh letters, although might also create an additional confusion due to phonological decoding as it was previously demonstrated in relation to word reading^40–43^. And since phonological decoding is also an important process during writing (phonemes-to-graphemes), the degree of incongruency across Cyrillic Kazakh and Latin Kazakh alphabets might affect the handwriting performance in a second alphabet, both at a motoric and a linguistic level.

Not many studies have investigated the impact of bilingual educational systems or digraphia on handwriting. Lebanon is an example of a country that has a bilingual educational system. In their study, Abizeid et al.^44^ discuss the consequences of simultaneous bigraphism for Lebanese primary school children who are exposed simultaneously to Latin and Arabic scripts. Using the BHK test, a comparison was made between the handwriting of these children and that of French children. The results showed that the BHK quality scores for Lebanese children were better for grade 1 (due to earlier start from age 5) but were significantly lower for grades 2–5 in comparison to the French children on this measure. The researchers explained these findings by considering the hypothesis that the competition between two obviously different graphic systems might induce interference of one on the other. Similar such interference does exist in language production: Bilinguals need to make a greater cognitive effort to facilitate the use of the target language^45,46^. Likewise, there might be a risk that acquiring two scripts simultaneously could be detrimental to the development of handwriting skills. The lack of existing answers to this question motivated this study.

In this paper, we conducted a study with 190 children aged 6–11 years old who were native speakers of the Kazakh language. The children were asked to copy a short text in both the Cyrillic and Latin alphabets onto a tablet. In the next sections, we present the analyses used to compare the children’s handwriting in the two scripts in various aspects of handwriting (static, kinematic, pressure, and tilt) as a function of age. One of the objectives of the analyses was to understand the different types of learning transfer appearing between the two alphabets in relation to grade level for the different aspects of handwriting. We also explored how the handwriting specificity found in one alphabet could be translated in the other alphabet. Finally, we wanted to show that our features were able to describe in the same way the handwriting quality in the two alphabets, highlighting the features independent of the writing content, and demonstrating the possibility of creating a cross-lingual model for the detection of handwriting difficulties.

Our results are then discussed to see what implications the change in alphabet may have for the education of children in Kazakhstan, especially in terms of how handwriting difficulties are affected by the transfer in alphabet.

## Results

### Absolute features difference

The descriptions of the features used in this study as well as their clinical interpretations with regard to handwriting can be found in the “Methods” Section of this article.

For every feature and for all grades (from 1st to 4th), a Wilcoxon test was conducted to detect if the distribution of a given feature in the Cyrillic alphabet is similar to the distribution of this same feature in the Latin alphabet. This statistical test was used since not all of the variables were found to follow a normal distribution. The results can be found in Table 1. In addition, the means and standard deviations for all distributions (by grade and alphabet) can be found in Supplementary Table S1.

**Table 1:** Feature differences between the two alphabets (Cyrillic and Latin) across grades.

		Grade 1		Grade 2		Grade 3		Grade 4	
Feature		W stat	*p* value	*W* stat	*p* value	*W* stat	*p* value	*W* stat	*p* value
Static									
Bandwidth Tremolo **- #1**		120	0.065	750	<5e-2	220	0.065	510	0.95
Median Tremolo **- #2**		100.0	<5e-2	630.0	<1e-3	290.0	0.069	530.0	0.41
Space Between Words **- #3**		110.0	<5e-2	390.0	<1e-3	130.0	<1e-3	430.0	0.076
Handwriting Moment **- #4**		70.0	<1e-2	730.0	<1e-2	280.0	<5e-2	420.0	<5e-2
Handwriting Density **- #5**		200.0	0.79	1300.0	0.97	220.0	<1e-2	430.0	0.074
Kinematic									
Mean Velocity **- #6**		200.0	0.63	980.0	0.088	210.0	<1e-2	320.0	<1e-2
Max Velocity **- #7**		110.0	<5e-2	260.0	<1e-3	80.0	<1e-3	200.0	<1e-3
In-Air-Time Ratio **- #8**		38.0	<1e-2	420.0	0.92	140.0	0.84	150.0	0.24
Bandwidth Speed **- #9**		160.0	0.6	710.0	<5e-2	330.0	0.76	260.0	<5e-2
Median Speed **- #10**		160.0	0.24	810.0	<1e-2	280.0	<5e-2	270.0	<1e-3
Pressure									
Mean Pressure **- #11**		110.0	<5e-2	1200.0	0.5	390.0	0.58	440.0	0.094
Mean Speed of Pressure Change **- #12**		120.0	<5e-2	380.0	<1e-3	180.0	<1e-2	190.0	<1e-3
Max Speed of Pressure Change **- #13**		190.0	0.55	660.0	<1e-3	160.0	<1e-3	280.0	<1e-3
Nb of Peaks of Pressure Change per secs **- #14**		2.0	<1e-3	47.0	<1e-3	55.0	<1e-3	64.0	<1e-3
Median Pressure **- #15**		50.0	<1e-3	430.0	<1e-3	180.0	<1e-3	240.0	<1e-3
Tilt									
Std. of of Tilt-X **- #16**		140.0	0.082	280.0	<1e-3	150.0	<1e-3	200.0	<1e-3
Std. of Speed of Tilt-X Change **- #17**		130.0	0.056	720.0	<1e-2	200.0	<1e-2	210.0	<1e-3
Median Tilt-y **- #18**		120.0	<5e-2	630.0	<1e-3	310.0	0.12	470.0	0.17

For each feature, a Wilcoxon test was conducted to see if there was a statistical difference in the means of the two distributions.

Before presenting the interpretations of the results, it is important to note that the children involved in this experiment were bilingual from birth (Kazakh and Russian languages), attended a Kazakh-speaking school, and studied English as a foreign language for a few hours per week starting from grade 1. The study was conducted in Spring 2019 after the change in script had already been announced by the Kazakh authorities (February 2018) but before it was applied. Thus, none of the participating children had received any formal training in the new Latin-based Kazakh script. Within this context, we were able to replicate the conditions that will be present at the moment of transition (see Table 3).

Different types of features are noticeably different in their co-evolution across the grades within the two alphabets.

#### Transferable features affected by alphabet differences

**Fig. 2:**
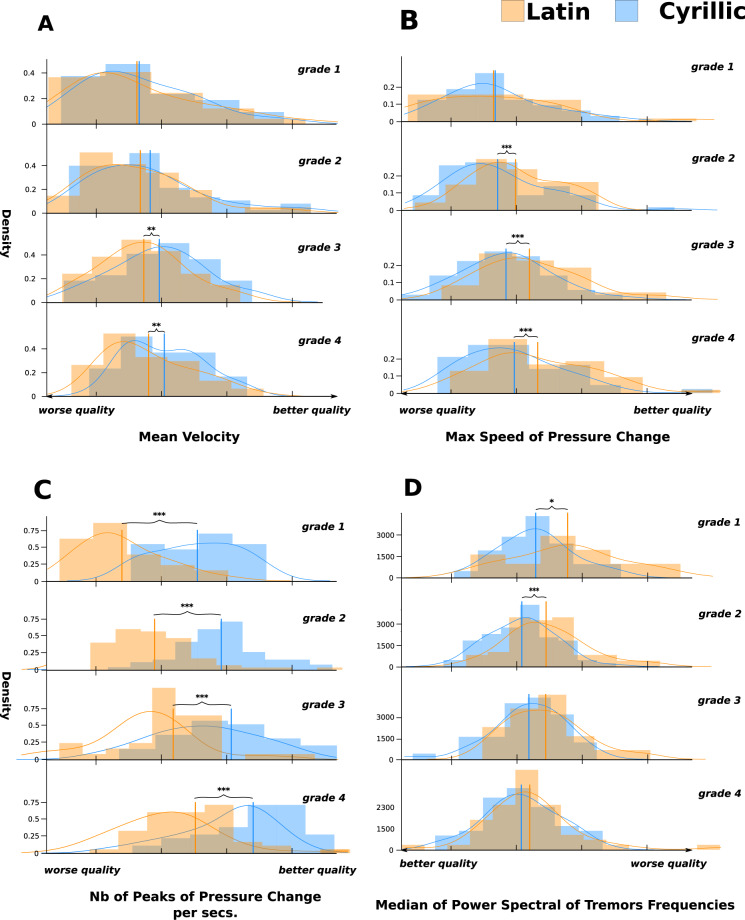
Distributions of the given features according to grade for the two alphabets (Cyrillic alphabet in blue, Latin alphabet in orange). The vertical lines represent the means of the distributions. **A** Mean Velocity feature. **B** Mean speed of the pressure change feature. **C** Nb of the peaks of pressure change per sec. **D** Median of the power spectral of tremor frequencies. The handwriting quality aspect along the *x*-axis was computed according to the Latin standard. Asterisks denotes statistical level of significance (*p < 0.05, **p < 0.01, and ***p < 0.001).

Significant differences that could stem from intrinsic differences between the two alphabets were observed. Since the learning time difference between the two alphabets increased by grade (see Table 3), features that are different for all the grades (across four developmental ages) fall into this category (a typical example of such a feature can be found in Fig. 2C). Three of the four pressure and kinematic features, as well as one static feature, were regrouped into this category.

The *Mean Speed of Pressure Change* (#12) and *Max Speed of Pressure Change* (#13) were found to be significantly higher when children were writing in the Latin alphabet compared to when they were writing in the Cyrillic alphabet across all grades (see Fig. 2B and Supplementary Table S1). These results are surprising since these features were found to be strongly inversely correlated with handwriting difficulties by Asselborn et al.^21^ (high values for these features can be translated into higher degrees of handwriting automation). The results found here appear to be abnormal since the mean scores for these features were higher when the children were writing in Latin, leading us to believe that the differences in these features were the result of intrinsic differences between the two alphabets. The same effect was found for the two most discriminative features used to diagnose handwriting difficulties in the Latin alphabet in Asselborn et al.’s study^21^: the distributions of the *Median of the Power Spectral of Speed Frequencies* (#10) and the *Bandwidth of the Power Spectral of Speed Frequencies* (#9) were found to be significantly different in Latin and Cyrillic for most of the grades, although the same abnormality was present in both (quality higher in Latin compared to Cyrillic if we read these features with the Latin model).

It is also very interesting to note the evolution of these features with grade. As illustrated in Fig. 2B, for both alphabets, a shift appears in the direction of handwriting proficiency with grade (the max speed of pressure change increases as children reach the higher grades). Even when there is an absolute difference between alphabets for this feature, its evolution follows the same path for both alphabets. In other words, this feature is still an indicator of handwriting automation for both alphabets even though a shift exists. This phenomena is shared with all the features described here.

Other pressure features seem to be affected by the difference in alphabet. The *Nb of Peaks of Pressure Change Per Sec* (#14) was found to be consistently higher when children were writing with the Cyrillic alphabet compared to when they were writing with the Latin alphabet, regardless of grade (see Supplementary Table S1). The *Median of the Power Spectral of Pressure Frequencies* (#15), a proxy for handwriting automation, was also found to be significantly different between the two alphabets whatever the grade. For these two features, however, no abnormalities were noted: If we interpret these features with the Latin model developed in Asselborn et al.^21^, then handwriting using the Cyrillic alphabet was found to be of higher quality. The *Maximum Velocity* (#7), always higher when children were writing in Cyrillic, can also be interpreted in the same manner (see Supplementary Table S1).

Two tilt features can also be sorted into this category. The *Std. of Tilt-X* (#16) and the *Std. of Speed of Tilt-X change* (#17) were always different between the two alphabets. Once again, children writing with the Cyrillic alphabet exhibited a higher standard deviation, which appears to be abnormal. In this sense, we believe that the difference comes from the alphabets requiring different pen manipulation styles (and thus pen tilts) during writing.

**Fig. 3:**
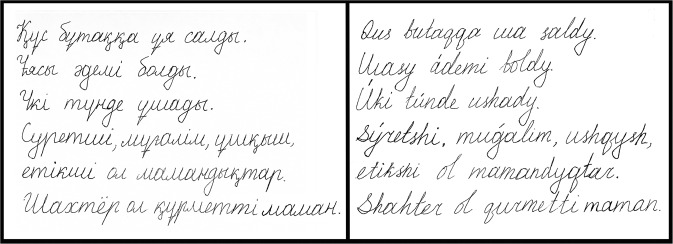
Two handwriting samples produced by the same person. The text written in the Cyrillic alphabet is on the left, while the text written in the Latin alphabet is on the right.

Finally, the *Space Between Words* (#3) feature was found to be consistently higher when children wrote in Cyrillic compared to Latin (see Supplementary Table S1). This feature computes the mean distance between strokes, which, in general, is the distance between words. In the case in which a word is composed of letters requiring writers to raise their pen in the middle, the distance traveled between the moment the pen rises and the pen touches the surface again is taken into account in the computation of this feature. We believe that the differences exhibited here signify more than an intrinsic difference between the two alphabets and that they are related to the difference in the learning times spent on each alphabet. Indeed, since our study provided their first opportunity to write in Latin, some children tended to write the letters one after the other with less continuity than they used when writing in Cyrillic (as can be seen in Fig. 3). Hence, there tended to be small distances between letters in words when writing with the Latin alphabet, which, when averaged, were responsible for the difference noted.

#### Feature with slow transfer between alphabets

Other features, such as the *Mean Velocity* (#6), presented interesting trends. Figure 2A displays the distributions for the *Mean Velocity* (#6) for the two alphabets for grades 1–4. Note that the mean of the distribution for the Cyrillic alphabet is always higher than the mean for the Latin alphabet (see Supplementary Table S1 for additional information). This difference appears to be a result of the difference in learning time between the two alphabets. We also see that the two distributions are shifting to the right (in the direction of handwriting proficiency) with grade (for both alphabet, a Mann–Whitney *U* test has been performed between the first and last grade and shows statistically significant differences between the distributions: for Latin, *U* (562), *p* < 0.05, for Cyrillic, U(565), *p* < 0.01), meaning that, in both alphabets, the children wrote faster and faster with grade, which is both an indicator and a direct consequence of the level of automation in their handwriting. Note also that the mean velocity is increasing more rapidly for the Cyrillic alphabet. We believe that this trend is related to the learning transfer between the two alphabets. This transfer appears to be incomplete; consequently, there is an increasing difference between the two distributions.

The *handwriting density* (#5) follows the same trend: in both alphabets, children’s writing became smaller and smaller with grade (an indicator of handwriting quality), but the difference between alphabets grew (children wrote significantly smaller in Cyrillic than in Latin in grade 3 and this tendency seems to be the same for the last grade even if no statistically significant results could be extracted in grade 4 (*p* = 0.074)).

#### Features which are transferable when the level of automation is higher

Some of the features exhibited decreasing differences between the two alphabets with grade. For instance, this trend can be seen in the two features describing shakiness: the *Bandwidth of the Power Spectral of Tremor Frequencies* (#1) and the *Median of the Power Spectral of Tremor Frequencies* (#2). As illustrated in Fig. 2D, the general trend show a decrease in the *Median of the Power Spectral of Tremor Frequencies* (#2) with grade, meaning that, for both alphabets, the average level of shakiness decreased with age, which is a direct consequence of the increasing level of automation children acquire. Interestingly, the difference between the two alphabets seems to decrease: children writing in Latin presented a significantly higher level of shakiness compared to when writing in Cyrillic in the lower grades (1st and second grade), while the difference was nearly non-existent by grade 4. Hence, the transfer between the two alphabets is in line with the level of automation. In other words, as automation increases, better control of the pen in one alphabet is beneficial for the other alphabet.

Other features seem to follow the same trend. Such is the case for the so-called *In-Air-Time ratio* (#8), a feature that was found to be highly correlated with handwriting problems in various studies conducted using the Latin alphabet^21^ as well as the Hebrew alphabet^24,47^. We can see that the *In-Air-Time Ratio* (#8) is always smaller for the Cyrillic alphabet (see Supplementary Table S1), which is a sign of a better knowledge of the Cyrillic alphabet by heart and of better motor program (memory) of the letters. Although the difference between the two alphabets was statistically significant for the lowest grade, no differences were found for subsequent grades.

The *Median of the Power Spectral of Tilt-y Frequencies* (#18) also follows the same trend. The median was always higher when the children were writing in Cyrillic (which is in line with the results of Asselborn et al.^21^). Moreover, we can see that the difference in the feature decreased with grade.

Finally, the *Handwriting Moment* (#4) also falls into this category. Children were writing in "straight lines" to a greater degree when writing in the Cyrillic alphabet, which appears to be a normal result (writing in a straight line is an indicator of handwriting quality). The difference in "straightness" between the two scripts was found to decrease with grade.

### Relative feature difference

In this Section, we want to assess if the change of alphabet brought handwriting difference relative to other children. We will no longer focus on raw data differences but rather on the relative differences between individuals. In other words, we are interested in investigating if children who presented a low value for one feature in one of the alphabets *relative to the other children* (of the study) still presented a low value for this same feature in the other alphabet *relative to the other children* (of the study).

In Table 2, the Spearman rank correlation is reported for all features and grades. The Spearman Rank correlation coefficient is based on the ranked values for each variable rather than the raw data. The correlation between two variables (e.g., feature X in Cyrillic and feature X in Latin) will be high when the observations have similar ranks (i.e., the relative position labels of the observations within the feature X: 1st, 2nd, 3rd, and so forth) in the two alphabets. In other words, if a child has a low (high) value for one feature compared to other children in Latin and still has a low (high) value of this same feature in Cyrillic, the Spearman rank correlation will be high.

**Table 2:** Feature difference between the two alphabets (Cyrillic and Latin) for different grades.

		Grade 1		Grade 2		Grade 3		Grade 4	
Feature		corr	pvalue	corr	*p* value	corr	stat	corr	*p* value
Static									
Bandwidth Tremolo **- #1**		0.011	0.96	0.029	0.81	0.3	0.059	0.014	0.93
Median Tremolo **- #2**		0.21	0.27	0.13	0.27	0.29	0.065	0.31	<5e-2
Space Between Words **- #3**		0.77	<1e-3	0.58	<1e-3	0.52	<1e-3	0.46	<1e-3
Handwriting Moment **- #4**		0.35	0.066	0.42	<1e-3	0.68	<1e-3	0.56	<1e-3
Handwriting Density **- #5**		0.73	<1e-3	0.75	<1e-3	0.73	<1e-3	0.74	<1e-3
Kinematic									
Mean Velocity **- #6**		0.72	<1e-3	0.72	<1e-3	0.69	<1e-3	0.69	<1e-3
Max Velocity **- #7**		0.4	<5e-2	0.55	<1e-3	0.37	<5e-2	0.34	<5e-2
In-Air-Time Ratio **- #8**		0.45	<5e-2	0.53	<1e-3	0.4	<1e-2	0.53	<1e-3
Bandwidth Speed **- #9**		0.52	<1e-2	0.53	<1e-3	0.22	0.17	0.53	<1e-3
Median Speed **- #10**		0.22	0.25	0.56	<1e-3	0.54	<1e-3	0.4	<1e-2
Pressure									
Mean Pressure **- #11**		0.95	<1e-3	0.68	<1e-3	0.76	<1e-3	0.81	<1e-3
Mean Speed of Pressure Change **- #12**		0.67	<1e-3	0.57	<1e-3	0.37	<5e-2	0.54	<1e-3
Max Speed of Pressure Change **- #13**		0.84	<1e-3	0.55	<1e-3	0.69	<1e-3	0.54	<1e-3
Nb of Peaks of Pressure Change per secs **- #14**		0.68	<1e-3	0.5	<1e-3	0.47	<1e-2	0.37	<1e-2
Median Pressure **- #15**		−0.045	0.82	0.29	<5e-2	0.49	<1e-2	0.41	<1e-2
Tilt									
Std. Tilt-X **- #16**		0.53	<1e-2	0.58	<1e-3	0.6	<1e-3	0.7	<1e-3
Std. of Speed of Tilt-X change **- #17**		0.71	<1e-3	0.7	<1e-3	0.58	<1e-3	0.7	<1e-3
Median Tilt-Y **- #18**		−0.25	0.19	−0.012	0.92	0.22	0.16	0.37	<1e-2

For each feature, a Spearman’s rank correlation was calculated in order to examine the similarities between the two distributions.

In general, the features seem to be highly positively correlated between the two alphabets. Indeed, 90% of the correlations we computed were significant. This is a very interesting result since it shows that children are able to transfer their handwriting specificity from one alphabet to another. The implication of such a result will be discussed further in the Discussion. Note that the correlation levels increased with grade for the majority of the features. We believe that this result is a consequence of the increased level of automation shown by children as they age. Indeed, as they reach higher grades, their movements are more controlled, resulting in less variability in the way they write. In other words, a child in first grade will have a tendency to write the same letter differently every time (without the same pressure pattern, same speed, same acceleration and so on), while a child in fourth will always write it in the same manner. In this sense, children are able to transfer their motor skills from one alphabet to the other, i.e., a specific pattern of pressure used to write one shape in an alphabet will be used to write a similar shape in another alphabet.

## Discussion

In this study, we found a significant absolute difference between the Latin and Cyrillic alphabets for the majority of the features across four grades. We were expecting more similarities between the two languages than, for instance, in studies comparing Vietnamese to English or Chinese to English. Two types of features were used to describe handwriting: static features, which characterize the final graphical traces of handwriting, and dynamical features, which describe the kinematics, pressure and tilt aspects of handwriting. The 18 features used in this study were all found to explain the quality of handwriting for the Latin alphabet^21^.

Interestingly, these differences of the features likely evolve over the years. Remember that, in the present study, children in grades 1–4, respectively, have had 0.5–3.5 years of Cyrillic practice while they all have had no experience with the Latin-based Kazakh alphabet. Figure 2 shows three different situations in the co-evolution of the handwriting in both alphabets over grades, indicating different forms of learning transfer.

- In quarter A of Fig. 2: The differences between features in Latin and Cyrillic increase between grades 1 and 4. We see an improvement of these features in both scripts, such as average handwriting velocity, but Cyrillic improves faster. Since there is an improvement in Latin across grades despite that the Latin alphabet is new for all grades, we may conclude there is some transfer of skill between scripts. However, since the improvement in Latin is slower than in Cyrillic, it is probably a partial or *slow transfer*.
- In quarters B and C of Fig. 2: The differences of some features are constant across grades, i.e., the features improve in both the Cyrillic and Latin alphabets in a similar fashion. This type of evolution is evidenced by most of the pressure and tilt features. The stability of the differences across grades probably originates from the intrinsic differences between the two alphabets. Since these features indicate an improvement in writing in Latin with age, despite the fact all children had the same experience in Latin in all grades, we may also conclude that a positive transfer of fine motor skills (controlling pressure and pen tilt) occurs between the two alphabets from Cyrillic to Latin.
- In quarter D of Fig. 2: The differences in some features decrease between grades 1 and 4 (A Mann–Whitney U test shows statistical differences between the first and the last grade for both alphabets (*p* < 0.05)). Handwriting improves with both scripts, but Latin seems to catch up with Cyrillic in terms of quality. This type of evolution is seen in, for example, two features that capture the shakiness of handwriting. Such results may indicate a transfer dependent on the level of handwriting automation: better control of the pen in Cyrillic is beneficial to Latin.

In summary, these results can also be interpreted as indicators of the transfer of fine motor control skills from Cyrillic to Latin. This transfer is important but not homogeneous, with different aspects of handwriting being transferred more or less rapidly. These results are in line with previous research^37^ showing that performance in copying a foreign script correlates with fine motor skills. As children of older ages gain more experience in manipulating the pen, they become more efficient in copying. Interestingly, as these features appear to evolve in a similar way across grades in both alphabets, the metrics we developed for measuring handwriting skills in Latin handwriting will probably remain valid for Cyrillic handwriting, even if this should be validated by a large scale study.

Our results also show that the majority of the features are correlated in term of rank across the two alphabets, meaning that a child presenting a low value for a feature (compared to other children) in one alphabet will also present a low value for this same feature in the other alphabet. This correlation can also be interpreted as an indicator of transfer: A specific pattern of pressure used to write one shape in an alphabet will be used to write a similar shape in another alphabet. Interestingly, this correlation means that a child suffering form severe difficulties in Cyrillic will likely experience similar difficulties in Latin.

This transfer is rather surprising since motor skills constitute a small subset of the skills involved in handwriting. Our results might indicate that the transfer of these motor skills is partly independent from other cognitive or linguistic skills that explain larger differences between languages^33,35^, and our results present deeper evidence. This partial independence may be due to the fact that our analysis does not give much attention to the final (static) shape of letters but essentially the dynamics of the handwriting process, especially low-level features computed on every pixel of the final letters. This leads us to believe that these low level features of dynamics might be transferable to more languages.

- The transfer is slower for some features than for others, indicating the need for a progressive transition. We do not claim the transfer is rapid and systematic.
- The relative similarity of the two scripts, as compared to other situations of bigraphism such as studies in Lebanon for French and Arabic languages. Latin could be taught as the direct transliteration of the Cyrillic script in contrast to multidimensional differences between Arabic and Latin scripts^44^.
- In the early years, handwriting skills may be more related to fine motor control that for older children for whom cognitive-linguistics skills would be more crucial to explain performances (our oldest participants were around 9 years old).
- Our study focused only on writing skills measured through a copying task. It would be problematic to generalize the results found in this study to other writing tasks such as spontaneous writing or dictation where other skills are involved. Our study, hence, does not predict global effects of a radical change in Kazakh culture.

The ultimate goal of our research is to detect and remediate dysgraphia in multiple languages. One interesting side effect of these findings is that, as the features used to analyze handwriting seem to be transferable from one alphabet to another, they could be used to diagnose handwriting difficulties in the Cyrillic alphabet in the same way in which they were used to diagnose handwriting problems in the Latin alphabet^21^. The handwriting model built to detect dysgraphia could be reusable across alphabets. We need to collect new datasets produced by children with equal handwriting exposure in Cyrillic and Latin. Under such conditions, we would hope to find that the majority of the features were similar between the two alphabets. From the remaining features, where the differences would come from, we could learn the function mapping of the differences between the two distributions. With this function, the model we use to diagnose handwriting difficulties could be transferred from Latin to Cyrillic, i.e., it could then be used to detect handwriting problems in the Cyrillic alphabet.

Previous work has demonstrated the existence of cross-scriptal phonological mediation that can influence both word recognition^43^ and word writing^48^ in secondary school students and adults. Such mediation should additionally be considered in models seeking to characterize writing across scripts. However, the extent to which this phonological mediation is important for children, whose word recognition and word writing skills are still developing in both L1 and L2, must be tested in future studies. For example, the digraphic population would be an interesting pool of learners to investigate developmental aspects of handwriting.

## Methods

### Participants

A sample of 200 children was recruited from local primary schools in the capital of Kazakhstan. The children came from diverse socioeconomic backgrounds and were all native Kazakh speakers and writers. The children were 6–11 years old (*M* = 8.48, SD = 1.2). Out of the 200 children who volunteered, data for ten children could not be recorded due to a mishandling of the hardware and thus could not be included in the analysis, yielding a total of 190 (90 boys and 100 girls) valid and complete observations. The remaining children were from four grades: 29 children were first graders, 71 students were in grade 2, 41 children were in third grade and 49 students were in grade 4. Table 3 presents an overview of the sample of participating children.

**Table 3:** Summary statistics for the participants involved in this study.

	Grade 1	Grade 2	Grade 3	Grade 4
Male/female	13/16	33/38	21/20	23/26
Age (std.) [years]	6.94 (0.4)	7.94 (0.4)	8.88 (0.4)	10.16 (0.6)
Right handed/left handed	27/2	68/3	37/4	46/3
Cyrillic average learning time [months]	6	18	30	42
Latin average learning time [months]	0	0	0	0

Learning pace was 6 hours per week for handwriting classes in Cyrillic alphabet and 2 hours per week for English classes (English as a foreign language).

### Ethics statement

This research was approved by Nazarbayev University’s ethics committee. Informed consent was obtained in writing from all children and their parents. Supporting information included an assent form for children and an informed consent form for parents or guardians. Children received a brief explanation of the purpose of the study and the procedures involved in data collection. Assent and consent forms were distributed to children in their classrooms in the presence of their teachers. Children were asked to show the forms to their parents at home and submit them to their teachers, who then collected the forms for us during the days that followed.

### Data collection

Each child was taken outside of their classroom for approximately fifteen minutes and taken to an empty classroom. It was explained that they were to write on a Wacom Cintiq Pro 13 tablet using its stylus and that the task was to copy the text on a piece of paper that was positioned in front of them. Although previous work showed that a tablet stylus does not have an affect on writing performance^49,50^, prior to the start of the copying task, we asked children to write a few letters in order to get familiar with writing using a stylus. There were two versions of the same five sentences, i.e., one in each script (see Fig. 3). The text was selected to include diverse letters, in particular many specific Kazakh characters that are not present in English. The Cyrillic version of this text was taken from the alphabet learning textbook for children and then transferred to a Latin version.

A data collection tool was used to save the children’s demographic information (age, gender, grade, laterality) and the two handwritten texts. As children finished writing the first five sentences in one script, they were told to press the save icon and then write the next five sentences in another script.

The design of our experiment replicated the conditions which will be present in 2025: at the moment of data collection, children had spent different amounts of time practicing handwriting across grade levels. Children in grade 1 had spent approximately 6 months learning to write in Cyrillic script. For each subsequent grade level, we add an additional year of experience (12 months). Thus, children in grade 2 had spent approximately 18 months writing in Cyrillic, third graders had spent around 30 months writing in Cyrillic and fourth-graders had spent around 42 months (3 years and 6 months) writing in Cyrillic. The children practiced handwriting for 6 h per week, which started from simple shapes and moved to Cyrillic letters after approximately 6 weeks. All children had 2 h of English as a foreign language per week where they also started writing English letters in print version after approximately 6 weeks, and the learning time changed with grade level in a manner similar to the learning time for the Cyrillic script (i.e., 6 months of print handwriting in English for the first graders). However, the children had not been introduced to a cursive handwriting of English, therefore Latin-based Kazakh alphabet and its associated cursive handwriting were unfamiliar to children at the time of the study. Thus, in contrast to the Cyrillic script, the learning time for the new script was the same across all grade levels, i.e. it was equal to 0 (see Table 3).

### Features extraction

In previous work^21^, 53 handwriting features were defined and used to train a random forest classifier to diagnose dysgraphia. In this work, we only used the features that were found to be the most important in the aforementioned random forest model according to the Gini importance metric (averaged with a k-fold cross validation, *k* = 25). To maintain a good balance and compare the different groups of features, we selected the five most important features from each of the following four groups that we distinguished: static, pressure, kinematic, and tilt. Since only three features were found to be significantly different between children with and without handwriting difficulties, we decided to select only these three features for the tilt category. In the following paragraphs, we briefly provide their respective definitions. A more detailed description of these features can be found in^21^.

#### Static features

are purely geometric characteristics of the written text. Among the static features, we selected:

(1) and (2) The *Bandwidth of the Power Spectral of Tremor Frequencies* and the *Median of the Power Spectral of Tremor Frequencies* were included. Here, the tremors present in the handwriting of children can be noted for a given packet of points and can thus be described as a series. By doing so, we can apply the usual time series analysis and, in particular, the Fourier transformation and take the median of the resulting spectral distribution (as can be seen in 4). What we can observe from these two features is that children with handwriting difficulties show abnormal movements that translate into high frequencies in the Fourier transformation, resulting in a shift in the median towards higher frequencies. In the same manner, these children present a higher bandwidth since they are not consistent in the way they write.

(3) *Space Between Words* refers to the distance between strokes, which, in general, is the distance between words.

(4) *Handwriting Moment*. To compute this feature, we extracted bins of 300 points (from the same line of text) and computed their barycenters. The distance in the y direction between consecutive barycenters is computed and averaged for all of the points, reflecting the degree of straightness of the line of text written.

(5) *Handwriting Density*. A grid with 300-pixel cells covering the entire range of the handwriting trace is created. The number of points recorded by the Wacom tablet in each cell, if present, is then stored in an array. The mean of this array is represented by this feature. There is a positive correlation between handwriting density and handwriting quality, as handwriting becomes denser with age.

#### Kinematics features

regroup features describing the dynamics of the handwriting process. Among these features, we selected:

(6) and (7) *Mean Velocity* and *Maximum Velocity* quantify handwriting speed, where the speed is the distance traveled by the pen divided by the time taken. Research shows that children presenting handwriting difficulties have lower mean velocities as well as higher maximum velocities. Furthermore, mean velocity increases with age.

(8) *In-Air-Time Ratio*represents the proportion of time the writer spends without touching the surface of the tablet. This feature has been shown to be positively correlated with handwriting problems^21,24,47^.

(9) and (10) The *Bandwidth of the Power Spectral of Speed Frequencies* and the *Median of the Power Spectral of Speed Frequencies*. Handwriting can be interpreted as a two-dimensional time series. In the same manner as that for the *Median of the Power Spectral of Tremors Frequencies*, a Fourier transform can be calculated with the handwriting velocity and the median as well as the resulting bandwidth of the spectral distribution. We can observe very fast changes in speed in the handwriting of children with dysgraphia (some jerks resulting from a low level of handwriting automation). These abnormal changes in speed are translated into high frequencies in the Fourier transformation, resulting in a shift of the median towards higher frequencies. In the same way, children with a low level of automation change use variable speeds in their writing. Hence, a writer presenting a high bandwidth will not be fluent, as they are less consistent in their movements.

#### Pressure features

**Fig. 4:**
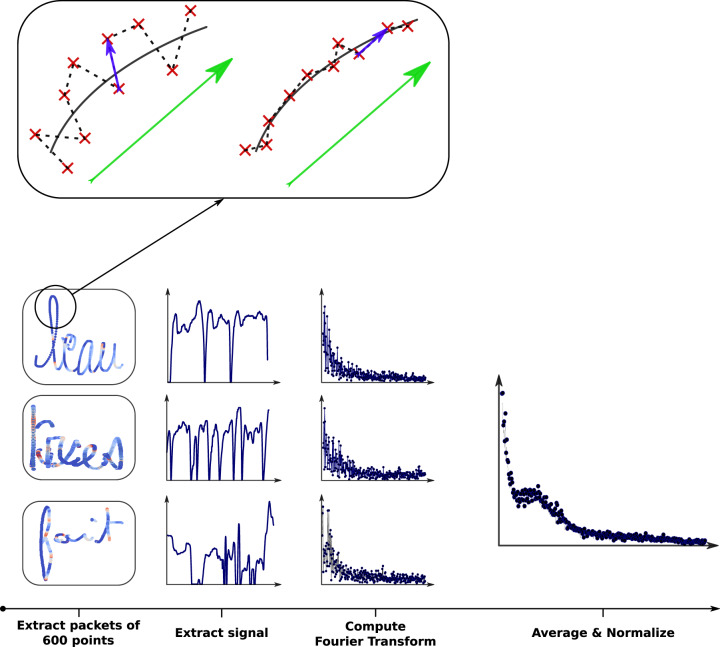
The whole process used to extract the frequency spectrum of our signal. (1) We first divided the BHK text into bins of 600 points. (2) For each bin, the signal was extracted. (3) We then computed the Fourier transformation of each signal. (4) We took the average of all signals and finally performed a normalization. At the top of the figure there is an example of a signal extracted from the data: the red dots are the point coordinates recorded by the device during handwriting. The vectors in blue are “local'” vectors linking two consecutive points. The vector in green is the “global” vector (average of the nine blue vectors) representing the global direction of the handwriting. The cross product of these two vectors gives us an indication of the smoothness/shakiness of the handwriting. The image on the right comes from a writer with smoother/less shaky handwriting than the one on the left. The cross product operation will detect this difference. The figure comes from^21^.

regroup features using the pressure measured between the pen tip and the tablet surface. Among these features, we selected:

(11) *Mean Pressure* is simply the average of all record points of pressure during writing.

(12) and (13) *Mean Speed of Pressure Change* and *Max Speed of Pressure Change* are extracted by working with averaged bins of 10 recorded points of pressure and dividing the time spent by the difference between these two averaged bins of points. These feature are then computed by taking the mean and the max of all measurements. These features are strongly positively correlated with handwriting proficiency: a high score for these features is a strong indicator of handwriting proficiency. Furthermore, we can see that the mean and the max speed of pressure change increase with age.

(14) *Nb of Peaks of Pressure Change Per Second* computes the number of pressure inversion per second. It is positively correlated with handwriting proficiency: the more inversion of pressure there is per second, the higher the handwriting proficiency is. This feature also increases with grade.

(15) *Median of the Power Spectral of Speed of Pressure Change Frequencies*. The speed of pressure change can be seen as a time series, and frequencies can be extracted using a Fourier transformation. The same process as that described in Fig. 4 is followed to extract the *Median of Speed of Pressure Change Frequencies*. In the same manner, children with a low level of automation change the pressure with they apply their pens inconsistently. For that reason, a high bandwidth is an indicator of low handwriting quality.

#### Tilt features

**Fig. 5:**
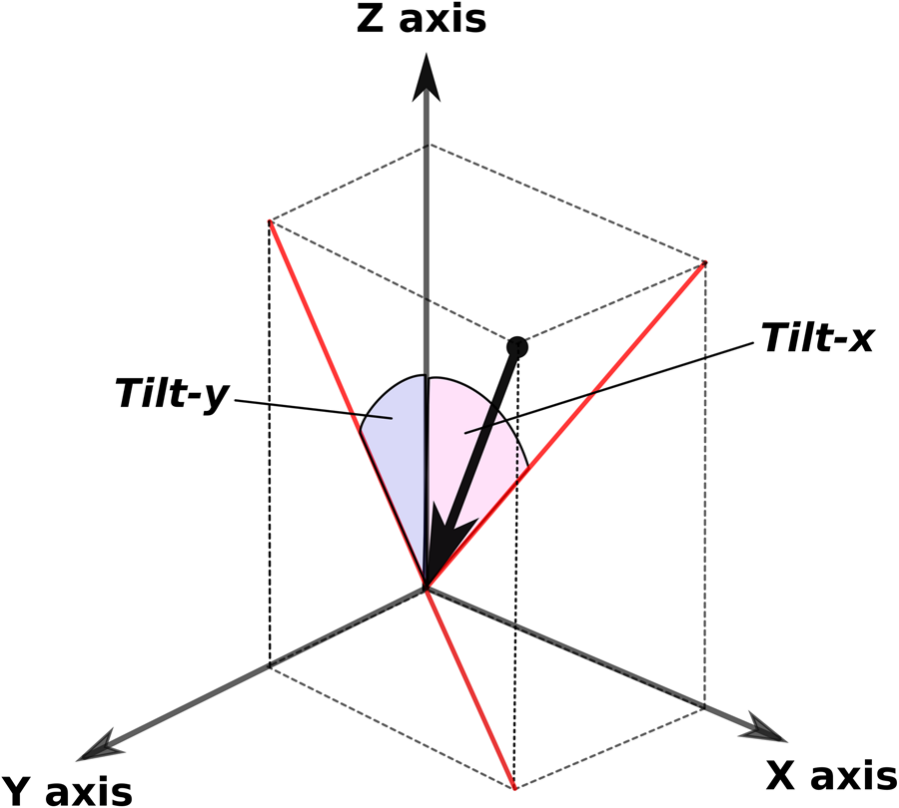
The two angles (tilt-x and tilt-y) recorded for the pen. The black arrow represents the pen, and the red segments represent its projections onto the *XZ* and *YZ* planes, respectively. The figure comes from^21^.

regroup features using the notion of tilt between the pen and the surface of the tablet (see Fig. 5). The tilt-x reflects the inclination of the pen in the direction of the written line, and the tilt-y reflects the inclination of the pen below the written line. Among these features, we selected:

(16) *Standard Deviation of Tilt-X Frequencies*. We can observe a more consistent control of the tilt-x in the pens of proficient writers and therefore a lower standard deviation for this group.

(17) *Standard Deviation of Speed of Tilt-X Frequencies*. We can see a lower *standard deviation of Speed of Tilt-X Frequencies* for proficient writers showing a more consistent control of their pen.

(18) *Median of the Power Spectral of Speed of Tilt-Y Frequencies*. Here, the Fourier transform of the two-dimensional time series can be calculated with the tilt-y logs as well as the median of the resulting spectral distribution (see Fig. 4). For the tilt-y, in contrast to what was observed for other categories of features, we can see that the non-dysgraphic children seem to exhibit higher frequencies during their handwriting, probably due to more constraining and rigid pen grips.

### Reporting summary

Further information on research design is available in the Nature Research Reporting Summary linked to this article.

## Supplementary information

Supplementary Table 4

Reporting Summary Checklist

## Data Availability

The data that support the findings of this study are available from the corresponding author upon reasonable request.

## Code availability

The code is available from the corresponding author upon reasonable request.

## References

[1] Graham S (1990). The role of production factors in learning disabled students’ compositions. J. Educ. Psychol..

[2] Berninger VW (1997). Treatment of handwriting problems in beginning writers: transfer from handwriting to composition. J. Educ. Psychol..

[3] Bourdin B, Fayol M (1994). Is written language production more difficult than oral language production? a working memory approach. Int. J. Psychol..

[4] Bourdin B, Fayol M (2000). Is graphic activity cognitively costly? A developmental approach. Read. Writ..

[5] Feder K, Majnemer A (2007). Handwriting development, competency, and intervention. Dev. Med. Child. Neurol..

[6] McCutchen D (2011). From novice to expert: implications of language skills and writingrelevant knowledge for memory during the development of writing skill. J. Writ. Res..

[7] Graham S (1990). The role of production factors in learning disabled students’ compositions. J. Educ. Psychol..

[8] Berninger V (1997). Treatment of handwriting problems in beginning writers: transfer from handwriting to composition. J. Educ. Psychol..

[9] Bourdin B, Fayol M (1994). Is written language production more difficult than oral language production? A working memory approach. Int. J. Psychol..

[10] Ziviani, J. & Wallen, M. The development of graphomotor skills. In Hand Function in the Child: Foundations for Remediation (2nd edition), 217–236 (A. Henderson & C. Pehoski, St. Louis, MO, 2006), mosby edn.

[11] Accardo A, Genna M, Borean M (2013). Development, maturation and learning influence on handwriting kinematics. Hum. Mov. Sci..

[12] Graham S, Berninger V, Weintraub N, Schafer W (1998). Development of handwriting speed and legibility in grades 1–9. J. Educ. Res..

[13] Charles, M., Soppelsa, R. & Albaret, J.-M. BHK: échelle d’évaluation rapide de l’écriture chez l’enfant (Paris, 2003), ecpa edn.

[14] Smits-Engelsman B, Niemeijer A, van Galen G (2001). Fine motor deficiencies in children diagnosed as DCD based on poor grapho-motor ability. Hum. Mov. Sci..

[15] Feder KP, Majnemer A (2007). Handwriting development, competency, and intervention. Dev. Med. Child Neurol..

[16] Christensen, C. A. The critical role handwriting plays in the ability to produce high-quality written text. The SAGE Handbook of Writing Development. pp. 284–299 (2009).

[17] Barnett A, Henderson S, Scheib D, Schulz J (2009). Development and standardization of a new handwriting speed test: the detailed assessment of speed of handwriting. Br. J. Educ. Psychol..

[18] de Ajuriaguerra, J., Auzias, M. & Denner, A. L’écriture de l’enfant (Neuchâtel, CH, 1971), delachaux et niestlé edn.

[19] Hamstra-Bletz, L., de Bie, J. & den Brinker, B. Concise evaluation scale for children’s handwriting. (Lisse, 1987), swets 1 zeitlinger edn.

[20] Zolna, K. et al. The dynamics of handwriting improves the automated diagnosis of dysgraphia. arXiv preprint arXiv:1906.07576 (2019).

[21] Asselborn T (2018). Automated human-level diagnosis of dysgraphia using a consumer tablet. npj Digit. Med..

[22] Asselborn T, Chapatte M, Dillenbourg P (2020). extending the spectrum of dysgraphia: a data driven strategy to estimate handwriting quality. Sci. Rep..

[23] Mekyska J (2016). Identification and rating of developmental dysgraphia by handwriting analysis. IEEE Trans. Hum.-Mach. Syst..

[24] Rosenblum S, Dror G (2016). Identifying developmental dysgraphia characteristics utilizing handwriting classification methods. IEEE Trans. Hum.-Mach. Syst..

[25] Usanova, I. Biscriptuality: Writing Skills Among German-Russian Adolescents, vol. 8 (John Benjamins Publishing Company, 2019).

[26] DeFrancis J (1984). Digraphia. Word.

[27] Altynsarina, E. Kazakhstan adopts new version of latin-based kazakh alphabet. (2018). https://astanatimes.com/2018/02/kazakhstan-adopts-new-version-of-latin-based-kazakh-alphabet/ Accessed on 1 July 2019.

[28] Unger, J. M. Functional digraphia in japan as revealed in consumer product preferences. Int. J. Sociol. Lang. pp. 141–152 (2001).

[29] Androutsopoulos, J. Greeklish’ Transliteration practice and discourse in the context of computer-mediated digraphia’. Orthography as Social action: scripts, spelling, identity and power. pp. 359–392 (2012).

[30] Allehaiby, W. H. Arabizi an analysis of the romanization of the arabic script from a sociolinguistic perspective. Arab World English J. 4 (2013).

[31] Planton S, Jucla M, Roux F-E, Démonet J-F (2013). The "handwriting brain”: a meta-analysis of neuroimaging studies of motor versus orthographic processes. Cortex.

[32] Li T, McBride-Chang C, Wong A, Shu H (2012). Longitudinal predictors of spelling and reading comprehension in chinese as an l1 and english as an l2 in hong kong chinese children. J. Educ. Psychol..

[33] McBride-Chang C, Chung KK, Tong X (2011). Copying skills in relation to word reading and writing in chinese children with and without dyslexia. J. Exp. Child Psychol..

[34] Wang Y, McBride-Chang C, Chan SF (2014). Correlates of chinese kindergarteners’ word reading and writing: the unique role of copying skills?. Read. Writ..

[35] Kalindi SC (2015). Beyond phonological and morphological processing: pure copying as a marker of dyslexia in chinese but not poor reading of english. Ann Dyslexia.

[36] Lam SS-Y, McBride C (2018). Learning to write: the role of handwriting for chinese spelling in kindergarten children. J. Educ. Psychol..

[37] Mo J, McBride C, Yip L (2018). Identifying the unique role of orthographic working memory in a componential model of hong kong kindergarteners’ chinese written spelling. Read. Writ..

[38] Beery, K. E. Beery vmi: the beery-buktenica developmental test of visual-motor integration. Minneapolis, MN: Pearson (2004).

[39] Zhexenova, Z. et al. A comparison of social robot to tablet and teacher in a new script learning context. Front. Robot. AI (2020). 10.3389/frobt.2020.00099 PMC7806116 33501266

[40] Lukatela G, Turvey MT (1998). Reading in two alphabets. Am. Psychol..

[41] Havelka J, Rastle K (2005). The assembly of phonology from print is serial and subject to strategic control: evidence from serbian. J. Exp. Psychol.: Learn. Mem. Cognit..

[42] Lukatela G (1999). Effects of frequency and phonological ambiguity on naming serbo-croatian words. Eur. J. Cognit. Psychol..

[43] Rastle K, Havelka J, Wydell TN, Coltheart M, Besner D (2009). The cross-script length effect: further evidence challenging pdp models of reading aloud. J. Exp. Psychol.: Learn. Mem. Cognit..

[44] Matta Abizeid C, Tabsh Nakib A, Younès Harb C, Ghantous Faddoul S, Albaret J-M (2017). Handwriting in lebanese bigraphic children: standardization of the bhk scale. J. Occup. Ther. Sch. Early Interv..

[45] Costa A, Sebastián-Gallés N (2014). How does the bilingual experience sculpt the brain?. Nat. Rev Neurosci..

[46] Green DW, Abutalebi J (2013). Language control in bilinguals: the adaptive control hypothesis. J. Cognit. Psychol..

[47] Rosenblum S, Parush S, Weiss PL (2003). The in air phenomenon: temporal and spatial correlates of the handwriting process. Percept. Motor Skills.

[48] Lukatela G, Turvey M (1990). Automatic and pre-lexical computation of phonology in visual word identification. Eur. J. Cognit. Psychol..

[49] Patchan MM, Puranik CS (2016). Using tablet computers to teach preschool children to write letters: exploring the impact of extrinsic and intrinsic feedback. Comput. Educ..

[50] Mayer C (2020). Literacy training of kindergarten children with pencil, keyboard or tablet stylus: the influence of the writing tool on reading and writing performance at the letter and word level. Front. Psychol..

