# Supplementary material for: The transferability of handwriting skills: from the Cyrillic to the Latin alphabet
Source: NPJ Sci Learn. 2021 Feb 23;6:6. doi: 10.1038/s41539-021-00084-w (PMC7902616; doi:10.1038/s41539-021-00084-w)
Supplement: Supplementary file 1 — Supplementary Table 4 [file 41539_2021_84_MOESM1_ESM.pdf]

Feature		Grade 1 Cyrillic	Grade 1 Latin	Grade 2 Cyrillic	Grade 2 Latin	Grade 3 Cyrillic	Grade 3 Latin	Grade 4 Cyrillic	Grade 4 Latin
Bandwidth Tremolo ***1e-2**	Static	2.53±0.07	2.56±0.06	2.53±0.04	2.55±0.05	2.54±0.04	2.56±0.04	2.54±0.03	2.54±0.04
Median Tremolo ***1e3**		2.73±0.12	2.83±0.17	2.68±0.11	2.76±0.13	2.71±0.12	2.76±0.13	2.68±0.12	2.71±0.15
Space Between Words ***1e3**		5.09±4.48	3.54±2.54	5.17±3.64	3.26±2.46	3.99±2.21	2.65±1.46	4.73±3.59	3.68±2.36
Handwriting Moment ***1e4**		5.83±8.26	14.2±12.3	7.89±10.7	12.3±12.3	8.03±10.1	10.2±11.0	7.28±7.05	9.50±8.53
Handwriting Density ***1e-2**		1.33±0.80	1.31±0.95	1.33±0.68	1.34±0.72	1.15±0.59	1.27±0.54	1.04±0.38	1.19±0.60
Mean Velocity	Kinematics	1.98±0.93	1.94±1.07	2.17±1.07	2.00±1.06	2.31±0.78	2.06±0.73	2.41±0.66	2.14±0.75
Max Velocity ***1e-1**		1.98±0.83	1.49±0.88	1.95±0.72	1.44±0.67	1.84±0.61	1.33±0.43	2.01±0.62	1.57±0.56
In-Air-Time Ratio ***1e1**		2.73±1.46	3.87±1.58	3.06±1.46	3.06±1.48	2.61±1.39	2.69±1.51	2.99±1.32	3.23±1.50
Bandwidth Speed ***1e-2**		2.62±0.03	2.63±0.04	2.62±0.02	2.61±0.03	2.62±0.03	2.62±0.03	2.62±0.02	2.61±0.03
Median Speed ***1e3**		2.58±0.13	2.69±0.28	2.56±0.10	2.55±0.19	2.58±0.10	2.59±0.24	2.57±0.09	2.54±0.18
Mean Pressure ***1e1**	Pressure	3.93±1.28	3.73±1.28	3.66±1.07	3.63±0.97	3.65±1.14	3.62±0.90	3.58±1.18	3.48±1.00
Mean Speed of Pressure Change ***1e1**		1.96±0.86	2.42±1.48	1.98±0.87	2.64±1.03	2.17±0.82	2.73±1.07	2.01±0.84	2.70±1.07
Max Speed of Pressure Change		3.78±1.35	3.73±1.76	3.85±1.38	4.36±1.22	4.09±1.26	4.75±1.38	4.32±1.48	4.99±1.60
nb Peaks of Pressure Change per secs		2.34±0.53	1.37±0.52	2.65±0.53	1.79±0.56	2.78±0.68	2.03±0.64	3.06±0.60	2.31±0.66
Median Pressure ***1e3**		2.54±0.09	2.70±0.18	2.56±0.10	2.63±0.11	2.59±0.09	2.66±0.15	2.54±0.11	2.61±0.14
Std tilt-X ***1e2**	Tilt	1.98±6.26	1.75±7.74	1.94±7.35	1.77±7.51	1.97±5.47	1.74±5.38	1.95±6.70	1.68±5.67
Std Speed of tilt-X change ***1e1**		1.98±0.63	1.75±0.77	1.94±0.73	1.77±0.75	1.97±0.55	1.74±0.54	1.95±0.67	1.68±0.57
Median tilt-Y ***1e3**		9.40±0.35	9.31±0.50	9.45±0.29	9.47±0.39	9.56±0.28	9.43±0.37	9.63±0.23	9.54±0.29

Supplementary Table 1. Feature means and standard deviations for the two alphabets (Cyrillic and Latin). For visibility purposes, some features values have been multiplied by the factor next the feature name.
